# Supplementary material for: The human cytomegalovirus chemokine binding protein UL22A is necessary for efficient reactivation from latency in CD34+ hematopoietic progenitor cells and humanized mice
Source: J Virol. 2026 Jul 27;100(8):e00612-26. doi: 10.1128/jvi.00612-26 (PMC13483475; doi:10.1128/jvi.00612-26)
Supplement: Supplementary file 1 [file jvi.00612-26-s0001.docx]

**SUPPLEMENTAL FIGURE LEGENDS**

**Figure S1. UL22A is necessary for reactivation from latency in primary fetal liver-derived CD34^+^ HPCs.**

Fetal liver-derived CD34^+^ HPCs were infected with TB40/E UL22AHF or UL22AmutHF at an MOI of 3 for 48 hours and then sorted using FACS for viable, CD34^+,^ GFP^+^ cells. Latency and reactivation experiments were performed as in Figure 2B.

**Supplemental Figure 2. UL22A tyrosine residues Y65 and Y69 are not necessary for lytic replication.**

NHDFs were infected with TB40/E UL22AHH, UL22AHH 2X STOP, UL22AHH Y65F, UL22AHH Y69F, and UL22AHH Y65F+Y69F at an MOI of 3 for single step (A and B) or an MOI of 0.01 for multistep (C and D) growth curves. Cell-associated and supernatant virus were harvested at the indicated timepoints, and PFU/mL values were quantified. Data represents three independent experiments.
