# Supplementary material for: The human cytomegalovirus chemokine binding protein UL22A is necessary for efficient reactivation from latency in CD34+ hematopoietic progenitor cells and humanized mice
Source: J Virol. 2026 Jul 27;100(8):e00612-26. doi: 10.1128/jvi.00612-26 (PMC13483475; doi:10.1128/jvi.00612-26)
Supplement: Supplementary file 2 [file jvi.00612-26-s0002.docx]

**SUPPLEMENTAL FIGURE LEGENDS**

**Supplemental Figure 3. Validation of RANTES neutralization during latency using α-RANTES antibody.**

(A) hESC-derived CD34^+^ HPCs were Mock-infected, infected with TB40/E UL22AHF or UL22AmutHF at an MOI of 2 for 48 hours and then sorted using FACS for viable, CD34^+,^ GFP^+^ cells. Sorted cells were put into latency culture as in Figure 2B. At 14dpi, supernatants were taken and RANTES concentration was quantified using the Human CCL5/RANTES ELISA Kit (R&D Systems). (B) Control IgG or α-RANTES antibody at the indicated amounts were added to 50pg recombinant human RANTES for 2 hours, then RANTES was detected using the Human CCL5/RANTES ELISA Kit (R&D Systems). (C) CEM cells resuspended in 37^o^C HBSS with 1uM Fura Red, AM for viability and incubated for 30 minutes in a 37^o^C water bath. Recombinant RANTES and anti-RANTES were incubated at indicated concentrations for 30 minutes prior to assay. Background calcium flux was recorded for 20 seconds, sample was removed while recording, and RANTES (+/- anti-RANTES) was added to the cell suspension and placed back on the cytometer for recording. Events were recorded for 120 seconds.

**Supplemental Figure 4. Adding 5ug/mL of anti-RANTES antibody during latency does not complement the UL22A mutant viruses.**

hESC-derived CD34^+^ HPCs were infected with TB40/E UL22AHH or UL22AHH Y65F+Y69F at an MOI of 2 for 48 hours and then sorted using FACS for viable, CD34^+,^ GFP^+^ cells. (A) Cells were treated with 5ug/mL of α-RANTES antibody or a control IgG and replenished again after 5 days while maintained in LTBMC in transwells above a murine stromal support to establish latent infection. (B) For 12 days, cells were maintained in LTBMC in transwells above a murine stromal support to establish latent infection. At 14 dpi, an equal number of cells were mechanically disrupted and seeded over a fibroblast monolayer to measure virus in latency culture (pre-reactivation) or cultured in cytokine-rich media containing 5ug/mL of α-RANTES antibody or a control IgG. Control IgG or RANTES neutralizing antibody were replenished after 1 week of reactivation. At 21 days post-plating, GFP^+^ wells were counted, and the frequency of infectious centers were determined by ELDA software.
